# Evaluation of cassette‐based digital radiography detectors using standardized image quality metrics: AAPM TG‐150 Draft Image Detector Tests

**DOI:** 10.1120/jacmp.v17i5.6008

**Published:** 2016-09-08

**Authors:** Guang Li, Travis C. Greene, Thomas K. Nishino, Charles E. Willis

**Affiliations:** ^1^ Department of Imaging Physics University of Texas MD Cancer Center Houston TX; ^2^ Radiation Services, Inc. Dover FL USA

**Keywords:** digital X‐ray detector, image quality control, nonuniformity, modulation transfer function, anomalous pixels, minimum signal‐to‐noise ratio, Total Quality Tool, Quality Assurance Process, TQT, QAP

## Abstract

The purpose of this study was to evaluate several of the standardized image quality metrics proposed by the American Association of Physics in Medicine (AAPM) Task Group 150. The task group suggested region‐of‐interest (ROI)‐based techniques to measure nonuniformity, minimum signal‐to‐noise ratio (SNR), number of anomalous pixels, and modulation transfer function (MTF). This study evaluated the effects of ROI size and layout on the image metrics by using four different ROI sets, assessed result uncertainty by repeating measurements, and compared results with two commercially available quality control tools, namely the Carestream DIRECTVIEW Total Quality Tool (TQT) and the GE Healthcare Quality Assurance Process (QAP). Seven Carestream DRX‐1C (CsI) detectors on mobile DR systems and four GE FlashPad detectors in radiographic rooms were tested. Images were analyzed using MATLAB software that had been previously validated and reported. Our values for signal and SNR nonuniformity and MTF agree with values published by other investigators. Our results show that ROI size affects nonuniformity and minimum SNR measurements, but not detection of anomalous pixels. Exposure geometry affects all tested image metrics except for the MTF. TG‐150 metrics in general agree with the TQT, but agree with the QAP only for local and global signal nonuniformity. The difference in SNR nonuniformity and MTF values between the TG‐150 and QAP may be explained by differences in the calculation of noise and acquisition beam quality, respectively. TG‐150's SNR nonuniformity metrics are also more sensitive to detector nonuniformity compared to the QAP. Our results suggest that fixed ROI size should be used for consistency because nonuniformity metrics depend on ROI size. Ideally, detector tests should be performed at the exact calibration position. If not feasible, a baseline should be established from the mean of several repeated measurements. Our study indicates that the TG‐150 tests can be used as an independent standardized procedure for detector performance assessment.

PACS number(s): 87.57.‐s, 87.57.C

## I. INTRODUCTION

Digital radiography (DR) has been widely adopted in the clinic in the recent years. A variety of clinical implementations of digital X‐ray detectors have been developed. Although some image quality control (QC) tools are available for DR, there is an absence of standardized metrics needed to ensure the constancy of digital detector performance.^(1)^

Therefore, the American Association of Physics in Medicine (AAPM) formed Task Group 150 (TG‐150) to address this issue. Its detector subgroup drafted a report for digital image detector testing and developed a set of recommended tests (E. Gingold, personal communication, July 2012). Automated analysis software had been previously developed in the MATLAB (MathWorks, Natick, MA) environment, and validated by two independent groups.^2^, ^3^, ^4^

However, at this early stage, there remains a lack of understanding of expected outcomes and challenges that will be encountered performing these tests. This study performed eight quantitative tests out of eleven recommended tests for DR (Table 1): detector response, signal, noise, signal‐to‐noise‐ratio (SNR), nonuniformity, anomalous pixels, correlated artifacts, and modulation transfer function (MTF). The other three tests are visual inspection, large signal capacity, and assessment of correlated image artifacts, or a contrast‐to‐noise ratio (CNR) test, which requires an additional test object. The results of the CNR test may depend on the test object because the aluminum step phantom recommended by the Task Group can be expected to affect the beam quality differently for different calibration beam conditions. This study also documents interferences that the authors experienced when implementing these tests with mobile DR systems and wireless DR rooms in direct comparison with each vendor's automated QC tests. This study is intended to improve our understanding of the proposed TG‐150 detector tests and their relationship to commercial QC metrics.

**Table 1:** Recommended tests by TG 150 (E. Gingold, personal communication, July 2012).

*Recommended Tests*	*Performed in this Study*	*Comments*
Visual Inspection	Yes	All quantitative analyses were performed only on images that passed the visual inspection test
Detector Response	Yes	
Signal Nonuniformity	Yes	
Noise Nonuniformity	Yes	
Signal‐to‐Noise Ratio Nonuniformity	Yes	
Minimum Signal‐to‐Noise Ratio	Yes	
Anomalous Pixels/lines	Yes	
Spatial Resolution	Yes	MTF Test Optional Alternative
Correlated Image Artifacts	No	s
Contrast‐to‐Noise Ratio	No	
Large Signal Capacity	No	
Geometric Accuracy	No	Recommended only for CR & CCD based systems
Ghosting/Lag	No	Recommended only for CR

## II. MATERIALS AND METHODS

### A. Tests with Carestream wireless detectors

Images were acquired using seven Carestream (Carestream Health Inc., Rochester, NY) DRX‐1C (cesium iodide, CsI) detectors with four new Carestream DRX‐Revolution and one Carestream retrofit (based on a GE AMX‐4, GE Healthcare, Milwaukee, WI) mobile DR systems. These detectors consist of 20×24 detector chips. Each detector chip has 128×128 detector elements producing images with a matrix size of 2560×3072. Three of the seven detectors (Detector 1, 6, and 7) were tested on the same DRX‐Revolution mobile unit. Detector 1 was the original detector of the mobile unit but was replaced by Detector 6 after Detector 1 was damaged. Detector 6 was then replaced by Detector 7 one month later when electronic problems developed with Detector 6. The DR detector (Detector 5) on the retrofit system is the same model as those supplied with the DRX‐Revolution systems, although its X‐ray tube and generator are different.

DRX detectors were calibrated according to manufacturer instructions using the built‐in calibration procedure:

1. Place the detector in the plastic tray provided by the manufacturer for calibration and testing.
2. Set the tray on top of a lead apron spread on the floor to exclude backscatter from the calibration.
3. Adjust the X‐ray tube to a source‐to‐image distance (SID) of 182 cm.
4. Center the detector in the light field and open the collimator to cover the entire detector.
5. Insert a filter consisting of a 0.5 mm copper sheet and a 1 mm aluminum sheet into the built‐in filter holder of the tube housing with the copper side facing the tube.
6. Run the automatic calibration procedure to calibrate the detector with four preset mAs stations at 80 kVp. The four mAs stations include the following: (i) a “calibration condition” (∼9 mAs), (ii) a high level exposure (∼18 mAs), (iii) an intermediate level exposure (2.8 mAs), and (iv) a low level exposure (∼0.71 mAs).

The mAs stations varied slightly for each unit depending on the specific tube output determined by service engineer during installation.

After the DRX detectors were calibrated, without repositioning the detectors and X‐ray tubes, four flat‐field images were acquired at the previously mentioned four mAs stations at 80 kVp to determine the detector nonuniformity, signal‐to‐noise ratio (SNR), detector response, and anomalous pixels. Images of a Type 53 bar pattern (Fluke Biomedical, Cleveland, OH), oriented in directions parallel and perpendicular to the anode‐to‐cathode (A‐C) axis, were also acquired at the “calibration condition” (~ 9 mAs, 80 kVp) for measurement of the MTF. The TQT test (DIRECTVIEW Total Quality Tool, Carestream) was then performed without repositioning the detector and X‐ray tube to exclude any effects from a change in geometry. TQT analysis was performed using built‐in software.

### B. Tests with GE wireless detectors

The TG‐150 tests were performed with four GE FlashPad detectors on two Discovery XR656 systems (GE Healthcare, Milwaukee, WI), one in a general radiographic room and the other in a chest radiographic room. The FlashPad detector consists of eight scan modules and eight data modules. Each module contains 256 scan or data lines (J. Sabol, personal communication, April 2016). Thus, the detector pixels are addressed by the readout electronics in 64 individual blocks, each 256 rows×256 columns. For consistency of analysis with the Carestream DRX detectors, we treated the FlashPad detectors as if they were composed of a 16×16 array of 128×128 detector elements, producing images with a total matrix size of 2022×2022. However, in our opinion, the underlying detector read‐out configuration should not affect our results as long as the images were analyzed in a consistent manner.

The cassette‐based wireless detectors are not integrated into the exposure stations in the general radiographic room, but are interchangeable between five possible exposure positions: the wall stand, the table Bucky, the tabletop, the cross‐table holder, or arbitrary positions such as for use with a gurney. However, the system maintains only three distinct calibration files for each individual detector for the first three positions: one for wall stand use, one for table Bucky use, and one for tabletop use. In addition to nonuniformities in gain and offset of detector elements, each calibration file also corrects for the exposure gradient that is presented to the detector from the heel effect under each of those specific conditions of exposure. The tabletop calibration is performed with the A‐C axis orientation similar to the table Bucky. The calibration file for the tabletop is then used for all the rest of the positions: tabletop, cross‐table, and arbitrary positions. However, for exams in the positions other than the wall stand and table Bucky position, the system has no control over the detector orientation. Hence, the detectors could be put at the reversed calibration position during the exams in the tabletop, cross‐table, and arbitrary positions.

Each GE FlashPad detector is calibrated using a 20‐mm aluminum filter at 80 kVp with the grid removed. However, the calibration SID varies for different detector positions (e.g., 180 cm for wall stand position, 100 cm for table Bucky position, and 127 cm for the cross‐table position) and therefore, the calibration mAs setting varies accordingly to compensate for the difference in the calibration SID to yield a similar exposure level to the detector. Thus, with the 20‐mm aluminum phantom in the field, the four flat‐field images for TG‐150 were acquired also with four mAs stations at 80 kVp: 1) the calibration mAs, 2) half of the calibration mAs, 3) a low exposure condition (<1 mAs), and 4) ∼ 180% of the calibration mAs. The high level exposure was performed at 180% instead of 200% because the detectors were partly saturated at 200%. In addition to the flat‐field images, two Type 53 bar pattern images were also acquired in two directions using the calibration mAs at 80 kVp, as were done with the Carestream DRX‐1C detectors.

Two sets of TG‐150 tests were performed on the table Bucky and cross‐table detectors in the general radiographic room before their detector calibrations. One set was performed in the detectors' usual position, and the other set was performed after these two detectors' positions were exchanged (between the table Bucky and cross‐table positions). Subsequently, without changing the detectors back to their usual positions, detector calibrations were performed on these two detectors, which made the exchange permanent. Then, a third set of TG‐150 tests was immediately performed on these two detectors after the calibrations, which tested the detectors in their “new” usual positions. The detectors in the wall stand of the general radiographic room and the dedicated chest room, however, were only tested in their usual positions without immediate detector calibrations.

The Quality Assurance Process (QAP, GE Healthcare) is performed weekly in our clinic and after detector calibrations by service engineers.^(5)^ The results of the QAP testing reside on the system temporarily. The results are automatically harvested remotely via ftp, parsed, and stored in a database for longitudinal analysis. The QAP data used in this study were extracted from the test reports closest to dates corresponding to the TG‐150 testing. The QAP test uses the 20‐mm Al filter for uniformity tests, but not for images of their Image Quality Signature Test (IQST) Phantom, from which the MTF was calculated.^(6)^

There was a difference in the time intervals between when the QAP was performed and the TG‐150 testing of the GE detectors, and this was not the case for the TQT and the TG‐150 testing of the Carestream detectors. This larger time interval may have resulted in larger differences between the QAP and TG‐150 measurements of the GE detectors than between TQT and TG‐150 measurements of the Carestream detectors. This was necessary because the GE units were all in clinical service, affording us less flexibility in scheduling testing and detector calibrations by vendor service engineers in the clinics. However, we ensured that there were no inconsistencies in the values reported in routine weekly QAP data before and after the TG‐150 tests; therefore, we do not expect this difference in time between testing affected our comparisons.

### C. Image quality metrics and image analysis

For‐processing images (aka “ORIGINAL DATA”), which had a linear response to exposure levels, were obtained for the TG‐150 tests. These images were harvested from the mobile systems directly through a built‐in system function with the linearization function enabled. The for‐processing images from the XR656 systems are minimally processed raw images that were first sent to Philips iSite Enterprise PACS (Picture Archiving and Communication System) and then downloaded. These for‐processing images were analyzed using MATLAB software previously validated and reported.^(2)^ Minimum SNR, global and local signal, noise and SNR nonuniformity, MTF, number of anomalous pixels, and detector response were measured from images acquired at the calibration mAs according to the definitions provided in the TG‐150 detector subgroup draft report, as described below (E. Gingold, personal communication, July 2012).

#### C.1 Regions of interest (ROI)

The image analyses for nonuniformity, anomalous pixels, and minimum SNR were based on ROI sampling techniques. The TQT used 128×128 ROIs in order to match the dimensions of the detector chips. For TG‐150 testing, we analyzed the GE images using 128×128 ROIs.^(7)^ Three JPEG image files automatically generated by QAP and stored on the GE acquisition console suggest that different dimensions may be used for different metrics and the specifications of these ROIs were provided by GE (Table 2, J. Sabol, personal communication, September 2015).

TG‐150 analysis of signal nonuniformity requires an array of ROIs to be defined across the flat‐field image. This study arranged the ROIs in a two‐dimensional array to cover the central 82.5% of the flat‐field image, which was similar to the ROI arrangement in the TQT and QAP.

**Figure 1:**
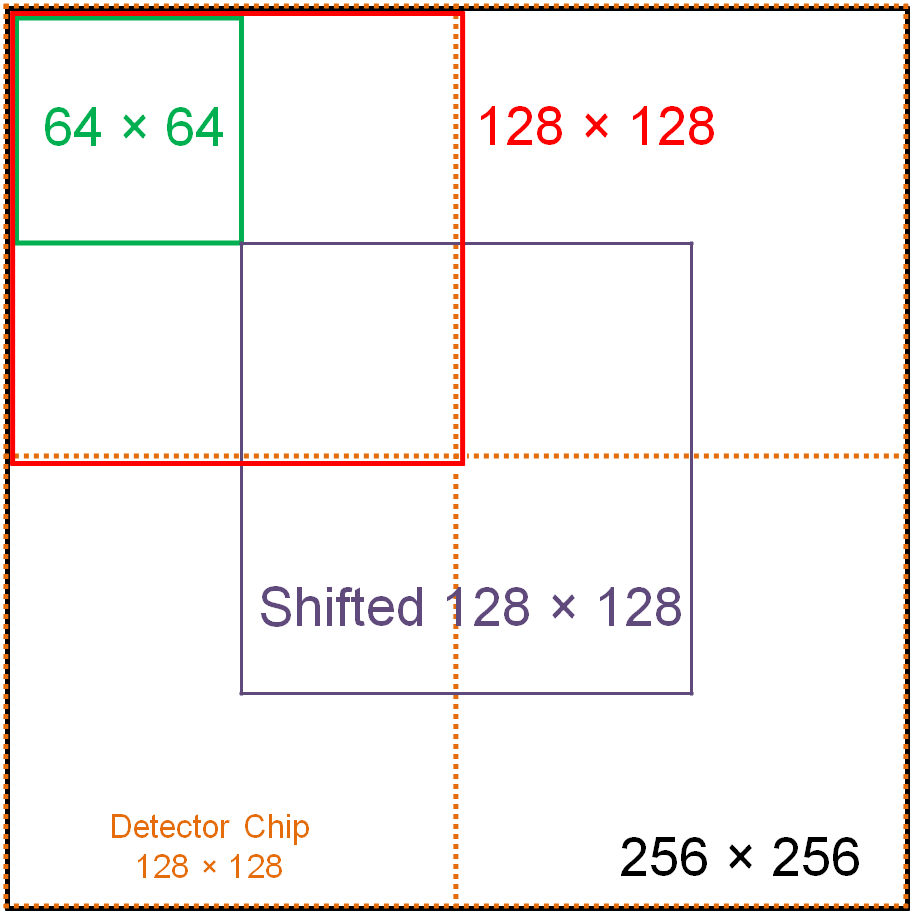
Four sets of regions‐of‐interest (ROIs) for the TG‐150 tests: 1) 128×128 ROIs (red line), aligned to the detector chips (green dotted line); 2) 256×256 ROIs (black dotted line), each including four adjacent 128×128 ROIs; 3) 64×64 ROIs (green line), four adjacent ROIs corresponding to a single 128×128 ROI; 4) shifted 128×128 ROIs (lavender line), with ROIs shifted by 64 pixels in both horizontal and vertical directions.

The effect of the ROI sizes on the TG‐150 image metrics was investigated using four sets of ROIs (Fig. 1) : 1) 128×128 ROIs matching the detector chips; 2) 256×256 ROIs, each including four adjacent 128×128 ROIs to include four detector chips; 3) 64×64 ROIs with four adjacent ROIs corresponding to a single 128×128 ROI; 4) shifted 128×128 ROIs, with 128×128 ROIs shifted by 64 pixels in both horizontal and vertical directions to straddle parts of four detector chips. All ROI sets excluded the detector chips on the edges, except for the shifted 128×128 ROI set which used half of the last column and half of the bottom row of detector chips.

**Table 2:** ROI used with QAP (J. Sabol, personal communication, September 2015).

*Quality Metric*	*ROI Matrix*	*ROI Size*	*ROI Overlap* ^a^	*Width of Excluded Image Border*
Local Brightness	37×37	50×50 pixels	0 pixels	75 pixels
Uniformity		(1×1 cm)	(0 cm)	(1.5 cm)
Global Brightness	24×24	150×150 pixels	75 pixels	75 pixels
Uniformity		(3×3 cm)	(1.5 cm)	(1.5 cm)
SNR Brightness Uniformity	34×34	150×150 pixels	100 pixels	100 pixels
Uniformity		(3×3 cm)	(2.0 cm)	(2.0 cm)

^a^ ROI overlap in both of the horizontal and vertical directions.

#### C.2 TG‐150 metrics of detector nonuniformity

The mean and standard deviation (SD) of pixel values in the i‐th ROI for each of the four sets of ROIs are denoted by μi and σi and the SNR of this ROI is denoted by ${\left(\frac{\mu }{\sigma }\right)}_{i}=\frac{\mu_{i}}{\sigma_{i}}$. Then, the following metrics were defined by TG‐150:

(1) $$\mathrm{Minimum SNR}=\min\left[{\left(\frac{\mu }{\sigma }\right)}_{i=1,2\ldots }\right]$$

(2) $$\mathrm{Global Signal Non}–\mathrm{uniformity}=\frac{\max(\mu_{i=1,2,\ldots })-\min(\mu_{i=1,2,\ldots })\;\mathrm{in all ROIs}}{\mathrm{mean}(\mu_{i=1,2,\ldots })}\times 100\%$$

(3) $$\mathrm{Global Noise Non}–\mathrm{uniformity}=\frac{\max(\sigma_{i=1,2,\ldots })-\min(\sigma_{i=1,2,\ldots })\;\mathrm{in all ROIs}}{\mathrm{mean}(\sigma_{i=1,2,\ldots })}\times 100\%$$

(4) $$\mathrm{Global SNR Non}–\mathrm{uniformity}=\frac{\max\left({\left(\frac{\mu }{\sigma }\right)}_{i=1,2,\ldots }\right)-\min\left({\left(\frac{\mu }{\sigma }\right)}_{i=1,2,\ldots }\right)\;\mathrm{in all ROIs}}{\mathrm{mean}\left({\left(\frac{\mu }{\sigma }\right)}_{i=1,2,\ldots }\right)}\times 100\%$$

(5) $$\mathrm{Local Signal Non}–\mathrm{uniformity}=\frac{\max[\max(\mu_{j,k=1,2,\ldots 8})-\mu_{j}]}{\mathrm{mean}(\mu_{i=1,2,\ldots })}\times 100\%$$

where $\mu_{j,k=1,2,\ldots ,8}$ are means of eight ROIs neighboring to the j‐th ROI

(6) $$\mathrm{Local Noise Non}–\mathrm{uniformity}=\frac{\max\left[\max\left(\sigma_{j,k=1,2,\ldots 8}\right)-\sigma_{j}\right]}{\mathrm{mean}\left(\sigma_{i=1,2,\ldots }\right)}\times 100\%$$

where $\sigma_{j,k=1,2,\ldots 8}$ are standard deviation of eight ROIs neighboring to the j‐th ROI

(7) $$\mathrm{Local SNR Non}–\mathrm{uniformity}=\frac{\max\left[\max\left({\left(\frac{\mu }{\sigma }\right)}_{j,k=1,2,\ldots 8}\right)-{\left(\frac{\mu }{\sigma }\right)}_{j}\right]}{\mathrm{mean}\left({\left(\frac{\mu }{\sigma }\right)}_{i=1,2,\ldots }\right)}\times 100\%$$

where ${\left(\mu /\sigma \right)}_{j,k=1,2,\ldots 8}$ are the SNR of eight ROIs neighboring to the j‐th ROI.

#### C.3 TG‐150 MTF

**Figure 2:**
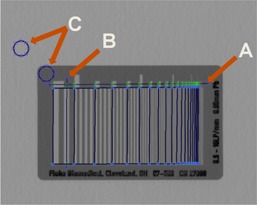
User interface that computes the MTF from a Type 53 bar pattern.

An interactive MATLAB subroutine was created^(2)^ to position an ROI over each bar pattern group by having the user designate two lines on the image. One line is drawn along the group indicators of bar pattern (arrow A in Fig. 2) and the other is drawn across the pattern group (arrow B in Fig. 2). The user then positions two circular ROIs to calculate the mean signal behind the pattern (0% modulation) and outside the pattern (100% modulation; arrow C in Fig. 2).

The variance of each bar pattern ROI was translated into discrete values of the MTF by means of the square wave response.^(8)^ This method is reported to be valid down to one‐third of the cutoff frequency, $f_{c}$ (3.60 lp/mm for the DRX‐1C detector). A continuous function was fitted to the MTF values using the Logit method and a weighted least‐squares fit.^(9)^ However, since the TQT and QAP only reports MTF at certain frequencies, this study only reported MTF with the bar pattern method at the corresponding frequencies.

#### C.4 Definition of anomalous pixels in TG‐150

Anomalous pixels were defined as pixels such that $p_{i,j}-\mu_{i}\geq 3\cdot \sigma_{i}$ in all four flat‐field images, where $p_{i,j}$ is the j‐th pixel in the i‐th ROI in the TG‐150.^2^, ^3^ Anomalous pixel analysis was designed to find the pixels that were not identified or adequately corrected by the automated gain and offset calibration and dead pixel correction software.

#### C.5 Calculation of detector response in TG‐150

Detector response in TG‐150 is characterized by a simple linear regression of mean pixel values of all sampled pixels of four flat‐field images on four mAs stations used to acquire corresponding images. The detector response is usually linear when the “for‐processing” images are used, although it may be log‐linear depending on how the images are obtained from these DR systems, and for other CR and DR systems. There are consequences for the values of metrics obtained from data that is log‐linear in the exposure domain.^10^, ^11^ After the logarithmic transform, the distribution of the signal response is altered. However, the signal noise should be inversely proportional to the square root of the exposure.^(10)^ The detector response reported in this study is the slope of the linear regression, which characterizes the detector sensitivity.

#### C.6 TQT image quality metrics

Based on 128×128 ROIs that match the detector chips, the TQT reports minimum SNR, as well as global nonuniformity metrics for signal, noise, and SNR with the flat‐field images. However, TQT does not report local nonuniformity measurements. TQT measures MTF using the edge method^(12)^ instead of the bar pattern method, and reports MTF in two directions perpendicular and parallel to the A‐C axis at 1 lp/mm,2 lp/mm,50% and 95% Nyquist frequency (1.80 lp/mm and 3.41 lp/mm, respectively)

TQT reports values for “Normalized Defects,” a proprietary metric, which is a weighted combination of several metrics for detector defects. It serves the same purpose as the analysis of anomalous pixels in TG‐150, but the two values are not directly comparable.

TQT also includes contrast‐to‐noise ratio (CNR) and dark noise measurements. In TQT, CNR values are measured with multiple low‐contrast patches included in its image quality phantom. CNR test was recommended by TG‐150 but was not included in our limited implementation. Dark noise testing was discussed, but not recommended by TG‐150.

#### C.7 QAP image quality metrics

The QAP software and its associated IQST phantom have undergone an evolution since described by Belanger et al. in 2004.^(6)^ The original version of the QAP reported 17 metrics, as shown in Table 3, including Dynamic Range measurements from the image of a step‐wedge, CNR measurements from the image of contrast patches, and resolution nonuniformity from the image of a uniform mesh. Noise power spectrum was also calculated and stored in a JPEG file. The original version was present on the XQi and XRd systems included in our original study.^(2)^

Although the features in the IQST remained the same, the QAP software with the Definium systems stopped reporting the six metrics associated with dynamic range, CNR, and resolution nonuniformity, added an overall pass or fail result, and relaxed some of the performance limits.

With the introduction of the Discovery systems, the features necessary for the dynamic range, CNR, and resolution nonuniformity measurements were removed from the IQST phantom. The QAP for wireless systems now reports pass or fail results for “ARC analog and digital” tests, where ARC is an acronym for Apollo Readout Chip, an application‐specific integrated circuit, and Apollo is a name given to its flat‐panel X‐ray detectors by GE Healthcare. The QAP software no longer reports MTF measurements when performed in the tabletop position where the IQST is not used, as is the same for the GE Optima AMX220, their mobile wireless DR system. The system considers a detector in the cross‐table position to be tabletop; therefore, metrics for MTF are not reported for the cross‐table positions either.

**Table 3:** QAP image quality metrics.

	*GE Revolution XQi & XRd*	*GE Definium and XR656*	
*Model Position*	*Table, Wall stand*	*Table, Wall stand*	*Cross‐table, Tabletop*
Overall Result	×	√	√
Number of Bad Pixels	√	√	√
Local Brightness Nonuniformity	√	√	√
Global Brightness Nonuniformity	√	√	√
SNR Nonuniformity	√	√	√
Spatial MTF at 0.5 lp/mm	√	√	×
Spatial MTF at 1.0 lp/mm	√	√	×
Spatial MTF at 1.5 lp/mm	√	√	×
Spatial MTF at 2.0 lp/mm	√	√	×
Spatial MTF at 2.5 lp/mm	√	√	×
ARC‐Digital	×	√	√
ARC‐Analog	×	√	√
Elect. Noise	√	√	√
Correlated Noise	√	√	√
Dynamic Range ‐ level linearity	√	×	×
Dynamic Range ‐ level accuracy	√	×	×
Contrast/Noise Ratio 1	√	×	×
Contrast/Noise Ratio 2	√	×	×
Contrast/Noise Ratio 3	√	×	×
Resolution Nonuniformity	√	×	×

QAP reports numerical values of local and global brightness nonuniformity and SNR nonuniformity. “Brightness” refers to signal level and QAP SNR nonuniformity measures global SNR nonuniformity (J. Sabol, personal communication, September 2015). The QAP uses Eq. (2) to calculate the global signal (brightness) nonuniformity, but calculates the local signal nonuniformity and SNR nonuniformity differently. To calculate local signal nonuniformity, the QAP uses the equation:

(8) $$\mathrm{Local Signal Non}–\mathrm{Uniformity}=\frac{\max(\max(\mu_{j,k=1,2,\ldots 8})-\mu_{j},\;\mu_{j}-\min(\mu_{j,k=1,2,\ldots 8}))}{\mathrm{mean}(\mu_{i=1,2,\ldots })}$$

To calculate QAP SNR nonuniformity, the QAP uses Eq. (4) for global SNR nonuniformity. However, it uses the difference of two flat‐field images instead of a single flat‐field image as the basis for the measurement.

QAP uses the edge method to measure MTF^(12)^ and reports the average MTF of two vertical edges which are parallel to the A‐C axis at 0.5 lp/mm,1 lp/mm,1.5 lp/mm,2 lp/mm, and 2.5 lp/mm. QAP also reports the number of bad pixels but is an incremental measurement relative to the last gain and offset calibration. Therefore, it is not directly comparable to the total number of anomalous pixels assessed by TG‐150.

QAP also reports electric noise and correlated noise. Correlated noise and CNR are recommended by TG‐150 but not included in our limited implementation.

### D. Effect of positioning on TG‐150 metrics

With a cassette‐based detector used in a bedside application, unless the detector quality tests are performed immediately after the gain and offset calibration, there is limited coincidence between the exposure geometry during calibration and the geometry during testing. To determine the effects of uncertainty in positioning with respect to the A‐C orientation during the calibration, four sets of TG‐150 tests were performed on one detector using one of the Carestream Revolution systems in the following sequence:

1. Prepare to acquire images for Test Set 1. (Unknown calibration position with repositioning between repeated acquisitions); align the detector to the A‐C axis, but without the knowledge of the A‐C axis direction during the previous calibration.
2. Acquire four flat‐field images at the four mAs stations specified in the Materials and Methods section A. item 6.i.–iv. above.
3. Acquire two bar pattern images in directions parallel and perpendicular to the A‐C axis.
4. Reposition the detector and X‐ray tube while maintaining the detector orientation relative to the A‐C axis.
5. Repeat Steps 2 through 4 above four more times.
6. Prepare to acquire Test Set 2 (at the exact calibration condition without repositioning): calibrate the detector in a new position.
7. Repeat Steps 2 and 3 five times while maintaining the exact calibration position.
8. Prepare to acquire Test Set 3 (at the reversed calibration position without repositioning): rotate the detector 180° from the calibration orientation to the reversed calibration orientation. (The detector was rotated while holding down the plastic tray to immobilize it so that translational displacement of the detector was minimized).
9. Repeat Steps 2 and 3 in the reversed calibration position five times.
10. Prepare to acquire Test Set 4 (at the calibration condition with repositioning between repeats): return the detector to the calibration position.
11. Repeat Steps 2 through 4 five times.

### E. Interferences

Interferences from artifacts (structured noise, gradient, image lag, detector saturation, and collimator misalignment) and features in the periphery of the image (penumbra, scatter, and artificially filled‐in pixels) were encountered during our efforts to perform these tests in a clinical setting. They were observed using detectors from a variety of DR systems including the two cassette‐based systems in the present study and other integrated DR systems (Siemens Aristos FX Plus, GE Revolution XQi, GE Revolution XRd, and GE Definium 8000). These illustrate the range of interferences that may be encountered in practice and their impact on the TG‐150 metrics.

### F. Statistical analysis

Paired t‐tests were performed to compare the image metrics with 128×128 ROI to those of other sized‐ROIs. The reported p‐values were adjusted with Bonferroni correction^13^, ^14^ using Stata 11 (StataCorp LP, College Station, TX). Outliers were excluded according to Chauvenet's criterion.^(15)^

## III. RESULTS

### A. Effect of ROI size

**Figure 3:**
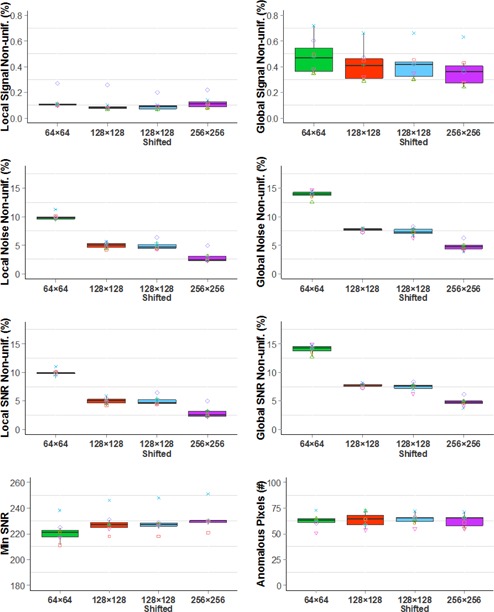
Boxplots of imaging quality metrics with 64×64 (green), 128×128 (red), shifted 128×128 (blue), and 256×256 ROIs (purple) for the TG150 tests. (Det1:◻,Det2:∘,Det3:Δ,Det4:+,Det5:×,Det6:⋄,Det7:∇).

**Figure 4:**
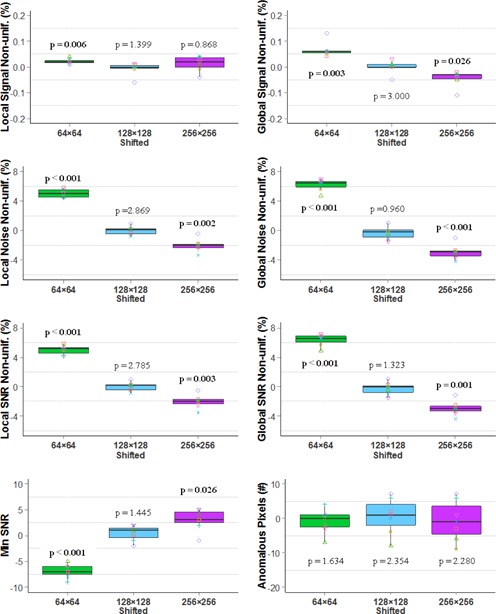
Boxplots of differences between the 128×128 ROI and the other ROIs, 64×64 (green), shifted 128×128 (blue) and 256×256 ROIs (purple) for the TG‐150 tests. P‐values were adjusted with Bonferroni correction. Bold adjusted p‐values indicate statistically significant. (Det1:◻,Det2:∘,Det3:Δ,Det4:+,Det5:×,Det6:⋄,Det7:∇).

Figure 3 shows the median and distribution of nonuniformity metrics, minimum SNR, and the number of anomalous pixels of all detectors. Figure 4 shows differences between these measurements with the 128×128 ROI and with the other ROIs. There were no statistically significant differences between the shifted 128×128 ROI and 128×128 ROI over all metrics, indicating well‐calibrated gain and offset. However, compared with the matching ROIs (128×128), the statistical analyses indicate that the nonuniformity measurements were higher using smaller ROIs (64×64) but lower using larger ROIs (256×256). The only exception was that there was no significant difference between 128×128 ROIs and the 256×256 ROIs for local signal nonuniformity, likely attributable to the large variance with the 256×256 ROIs. The analysis of minimum SNR values shows that minimum SNR was lowest with 64×64 ROIs, and highest with 256×256 ROIs. The number of anomalous pixels is unaffected by ROI size.

### B. Effect of positioning on TG‐150 metrics

**Figure 5:**
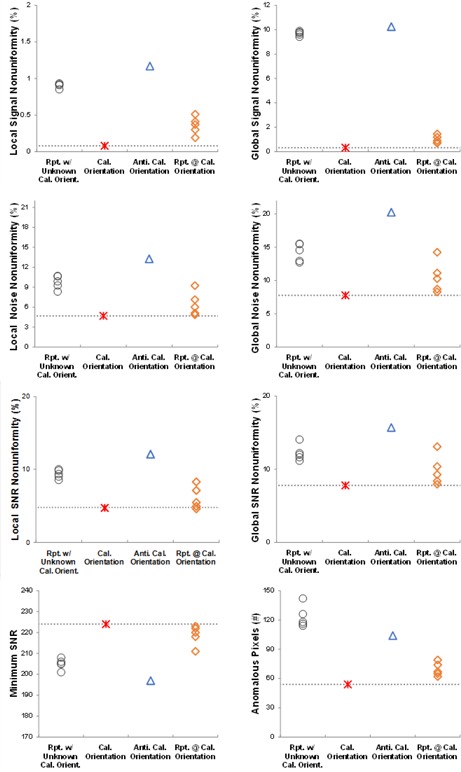
Repeated measurements of nonuniformity, minimum SNR, and anomalous pixels for the TG‐150 tests. Five sets of exposures repositioning X‐ray tube vs. detector (∘). After detector calibration, one set of exposures made without repositioning from calibration position (*). Detector rotated 180° from calibration orientation and another measurement made (Δ). Detector returned to calibration orientation and five more sets of exposures made with repositioning of tube vs. detector (◊).

Figure 5 shows the effect of positioning uncertainty on the TG‐150 metrics that is derived from ROI analysis of flat field images. The first group of repeated measurements, acquired without knowledge of the orientation of the detector during calibration, appeared similar to those obtained in the reversed calibration orientation. This suggests that the detector was likely aligned to the reversed calibration orientation during the first sequence. The second sequence of repeated measurements, acquired after the detector calibration, agreed with those obtained at the exact calibration condition, as expected. However, the second group of repeated measurements varied somewhat and was usually inferior to those acquired at the exact calibration condition.

**Figure 6:**
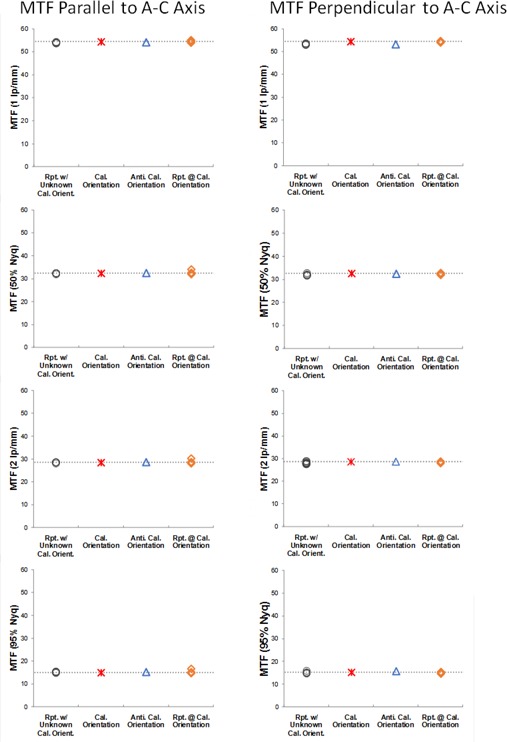
Repeated measurements of MTF by the TG‐150 test method. The images were acquired as described in Fig. 5.

Unlike the ROI based metrics derived from the flat‐field images that were highly sensitive to detector orientation and positioning, as reported by Dave et al.,^(4)^ the MTF test, which involves only small areas at the center of the images, appeared insensitive to detector positioning (Fig. 6).

The maximum absolute difference in MTF between the repeated measurements after calibration and the one in the exact calibration position was only 1.8%.

### C. Comparison of TG‐150 tests and TQT

TQT nonuniformity measurements were systematically higher than those of TG‐150 (Table 4), although they are similar. The two sets of values were not linearly correlated, which may be due to the fact that TQT uses a subset of 16×16 ROIs inside each of the 128×128 ROIs and the means of the subset ROIs are used to calculate mean and standard deviation of their parent 128×128 ROI, which results in a smaller variance than the mean and standard deviation of individual pixels. In addition to global nonuniformity measurements, TQT also reports minimum SNR which is linearly correlated with TG‐150 measurement ($R_{2}=0.76$).

**Figure 7:**
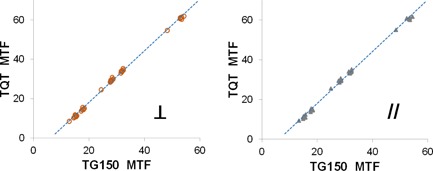
MTF measured by TQT versus MTF measured by the TG‐150 test method in orientations perpendicular and parallel to the A‐C axis. The MTF values measured by the two methods were strongly linearly correlated. (Perpendicular: ${\text{MTF}}_{\text{TQT}}=1.31\;\times \;{\text{MTF}}_{\text{TG}150}-8.16,R^{2}=0.9983$; Parallel: ${\text{MTF}}_{\text{TQT}}=1.31\;\times \;{\text{MTF}}_{\text{TG}150}-8.05,R^{2}=0.9976$)

TQT measures MTF using the edge method instead of the bar pattern method. Despite this difference in methodology, the values were highly correlated with an R‐squared value greater than 0.99 (Table 5 and Fig. 7).

**Table 4:** Comparison of TG‐150 and TQT metrics.

		*Det1*	*Det2*	*Det3*	*Det4*	*Det5*	*Det6*	*Det7*
*A) TG‐150 and TQT Common Metrics*								
minSNR	TG‐150	218	227	226	227	246	231	224
	TQT	220	234	228	229	238	234	227
Global Signal	TG‐150	0.45	0.3	0.29	0.41	0.66	0.47	0.32
Nonuniformity (%)	TQT	0.7	0.5	0.8	0.7	0.4	0.6	0.8
Global Noise	TG‐150	7.33	7.9	7.87	7.82	8.02	7.26	7.77
Nonuniformity (%)	TQT	9.5	8.2	9.7	9.7	10.6	8.1	10.3
Global SNR	TG‐150	7.33	7.77	7.85	7.92	8.18	7.3	7.79
Nonuniformity (%)	TQT	9.8	7.9	9.6	9.6	10.6	8	9.9
*B) TG‐150 Unique Metrics*								
Detector Response (${\text{mAs}}^{-1}$)		444.3	538.0	490.7	495.8	269.0	478.2	451.4
Characteristic Signal		3734	4512	4222	4178	3715	4573	4319
Characteristic Noise		16.62	19.1	17.96	17.82	14.56	19.05	18.52
Local Signal Nonuniformity (%)		0.08	0.08	0.07	0.07	0.1	0.26	0.08
Local Noise Nonuniformity (%)		4.22	5	4.51	5.14	5.64	5.42	4.68
Local SNR Nonuniformity (%)		4.17	5.1	4.5	5.13	5.83	5.46	4.78
Anomalous Pixels		64	64	72	59	72	59	54
*C) TQT Unique Metrics*								
Normalized Defects		1.33	1.215	0.993	1.984	2.048	1.261	N/R
CNR, Patch 1		4.66	4.95	4.8	4.92	5.68	4.92	5.21
CNR, Patch 2		3.75	4.02	4.07	3.85	4.69	3.93	3.98
CNR, Patch 3		2.39	2.58	2.64	2.56	2.93	2.48	2.38
CNR, Patch 4		1.63	1.7	1.58	1.56	1.78	1.56	1.59
CNR, Patch 5		1.11	1.24	1.14	1.12	1.32	1.03	1.08
CNR, Patch 6		0.61	0.63	0.69	0.68	0.71	0.63	0.57
Dark Noise Subtraction: Avg. ROI Signal Unif. (%)		0.6	0.5	0.8	0.7	0.8	0.6	0.8
Dark Noise Subtraction: Avg. ROI Noise Unif. (%)		11.2	10.3	12.2	10.9	18.6	10.7	12.9
Dark Noise Subtraction: Avg. ROI SNR Unif. (%)		11.3	10.6	12.7	10.8	19.1	10.9	13.1

**Table 5:** MTF from TG‐150 tests and TQT.

		*Det1*		*Det2*		*Det3*		*Det4*		*Det5*	*Det6*		*Det7*	
	*freq (lp/mm)*	*TG‐150*	*TQT*	*TG‐150*	*TQT*	*TG‐150*	*TQT*	*TG‐150*	*TQT*	*TG‐150*	*TQT*	*TG‐150*	*TQT*	*TG‐150*	*TQT*
	1	53.36	60.38	52.82	61.15	53.00	61.05	52.76	60.68	48.13	54.70	53.36	61.45	54.16	61.95
	2	27.57	28.40	27.74	29.33	28.26	29.58	28.14	29.15	24.33	24.55	28.61	29.98	28.29	30.80
	3	17.12	13.73	17.48	14.43	17.99	14.65	17.94	14.48	15.16	11.58	18.27	15.15	17.66	15.58
⊥	1.80 (50% Nyq)	31.43	33.05	31.51	33.95	31.98	34.15	31.85	33.73	27.79	28.68	32.35	34.60	32.19	35.35
	3.42 (95% Nyq)	14.43	10.28	14.81	10.70	15.30	10.90	15.27	10.88	12.82	8.53	15.56	11.35	14.91	11.80
	$R_{2}$	0.9991		0.9988		0.9989		0.9992		0.9994		0.9990		0.9980	
	1	53.45	60.23	52.30	61.03	53.19	61.03	53.06	60.53	48.38	55.18	53.41	61.25	54.37	61.78
	2	27.94	28.65	27.61	29.58	28.61	29.65	28.43	29.38	24.83	25.60	28.56	30.23	28.40	30.75
	3	17.50	13.88	17.50	14.68	18.33	14.78	18.16	14.63	15.64	12.53	18.20	15.25	17.70	15.53
//	1.80 (50% Nyq)	31.77	33.25	31.31	34.18	32.32	34.25	32.14	33.85	28.28	29.68	32.31	34.75	32.32	35.30
	3.42 (95% Nyq)	14.79	10.38	14.86	10.98	15.62	11.10	15.46	11.00	13.27	9.45	15.48	11.48	14.94	11.75
	$R_{2}$	0.9990		0.9986		0.9991		0.9991		0.9992		0.9986		0.9979	

### D. Comparison of TG‐150 tests and QAP

**Figure 8:**
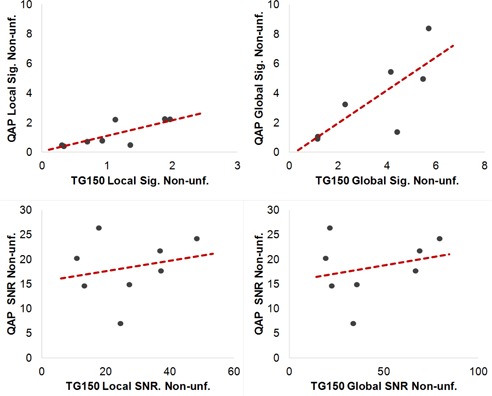
Simple linear regression between QAP and TG 150 nonuniformity measurements. One data point for local signal nonuniformity was excluded as an outlier according to Chauvenet's criterion. The local and global signal nonuniformity are correlated ($R_{2}=0.61$, 0.60, respectively) but not SNR nonuniformities ($R_{2}<0.07$).

Local and global signal non‐uniformities of TG‐150 were linearly correlated to those of QAP (Fig. 8, $R_{2}\approx 248;0.60$), when one data point for local signal nonuniformity metrics was excluded as an outlier according to Chauvenet's criterion, but their SNR nonuniformity metrics were not ($R_{2}\leq 0.07$). In addition to the lack of correlation, TG‐150 global and local SNR nonuniformity values decreased by 50%–60% after the detector calibration on Det2 and Det3, while in contrast, there was essentially no change in QAP SNR nonuniformity values before and after the calibration (Table 6). Therefore, it seems that TG‐150 SNR nonuniformity metrics are more sensitive to image nonuniformity compared to QAP's SNR nonuniformity metrics.

**Figure 9:**
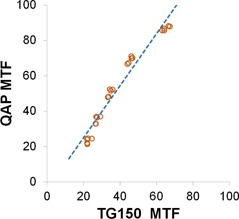
MTF reported by QAP versus MTF measured by the TG‐150 test method. The MTF values measured by the two methods were highly linearly correlated (${\text{MTF}}_{\text{QAP}}=1.47\times {\text{MTF}}_{\text{TG}150}-4.42,R_{2}=0.9552$).

As for MTF values (Table 7), the absolute differences between MTF values measured by TG‐150 and QAP were up to 24%, although they were linearly correlated (Table 7, $R_{2}>0.95$ and Fig. 9).

**Table 6:** TG‐150 and QAP metrics.

*Detector ID*	*Det1*	*Det2*			*Det3*			*Det4*
*Calibration Condition*	*No Cal*.^a^	*Before Cal*.		*After Cal*.	*Before Cal*.		*After Cal*.	*No Cal*.^a^
*QAP*								
Position Tested	Wallstnd	Table	X‐table	X‐table	X‐table	Table	Table	Wallstnd
Number of Bad Pixels	0	0	0	0	0	6	0	0
Local Brightness Nonuniformity	0.77	2.24	2.2	0.47	0.48	2.22	0.4	0.27
Global Brightness Nonuniformity	4.95	5.43	27.51	1.05	1.36	8.37	0.9	2.36
SNR Nonuniformity	7	21.74	14.89	14.63	17.68	24.22	26.39	7.47
Elect. Noise	2239	2205	3894	3855	4165	2409	2392	2379
Correlated Noise	7.34	10.92	4.29	4.32	5.87	12.46	9.81	8.37
*TG 150*								
Position Tested	Wallstnd	Table	X‐table	X‐table	X‐table	Table	Table	Wallstnd
Characteristic Signal	9446	9589	8234	9131	8589	9940	9892	8874
Characteristic Noise	35.28	44.45	34.21	33.98	34.5	44.54	36.72	32.2
Minimum SNR	217	113	194	241	132	106	242	245
Local Signal Nonuniformity (%)	0.93	1.89	1.13	0.31	1.36	1.97	0.34	0.7
Global Signal Nonuniformity (%)	5.48	4.16	3.64	1.17	4.42	5.71	1.16	2.29
Local Noise Nonuniformity (%)	26.32	81.69	32.21	12.69	81.03	111.06	17.48	12.55
Global Noise Nonuniformity (%)	38.62	114.81	40.2	23.06	111.1	139.73	21.01	21.08
Local SNR Nonuniformity (%)	24.57	36.93	27.34	13.31	37.21	48.39	17.82	10.96
Global SNR Nonuniformity (%)	33.86	68.96	35.76	22.49	66.79	79.49	21.48	19.28
Anomalous Pixels	594	843	504	397	418	890	242	499
Detector Response(${\text{mAs}}^{-1}$)	232.88	763.49	516.60	571.90	536.55	794.33	791.28	220.62

^a^ Calibration was not performed during the testing.

**Table 7:** MTF from the TG‐150 tests and QAP.

*Detector ID*		*Det1*	*Det2*				*Det3*					*Det4*
*Cal. Status*		*No Cal*.^a^		*Before Cal*.			*After Cal*.	*Before Cal*.			*After Cal*.		*No Cal*.^a^	
*Psn. Tested*		*Wallstand*		*Table*		*X‐table*	*X‐table*	*X‐table*	*Table*		*Table*		*Wallstand*	
	*Freq. (lp/mm)*	*TG‐150*	*QAP*	*TG‐150*	*QAP*	*TG‐150*	*TG‐150*	*TG‐150*	*TG‐150*	*QAP*	*TG‐150*	*QAP*	*TG‐150*	*QAP*
	0.5	67.61		64.71		74.42	65.17	73.90	63.81		64.82		66.63	
	1	47.35		46.01		50.94	46.26	48.11	43.90		44.53		47.38	
//	151.5	35.46		35.25		36.23	35.34	32.54	32.72		33.06		36.11	
	2	27.92		28.37		27.02	28.37	23.28	25.77		25.91		28.89	
	2.5	22.81		23.63		20.99	23.57	17.48	21.09		21.11		23.92	
	0.5	66.53	88.2	64.23	86.6	74.29	63.87	73.99	63.09	87.9	64.17	85.7	67.23	87.87
	1	46.12	71.1	46.25	70.0	50.79	45.00	48.91	44.01	70.1	44.71	67.3	46.81	70.05
⊥	1.5	34.33	52.4	35.88	52.2	36.10	34.28	33.61	33.29	51.6	33.68	48.2	34.91	51.6
	2	26.92	36.9	29.20	37.0	26.92	27.47	24.36	26.55	36.4	26.74	32.9	27.41	36.41
	2.5	21.93	24.0	24.55	24.5	20.91	22.82	18.49	21.97	24.7	22.03	21.4	22.33	24.65

^a^ Calibration was not performed during the testing.

### E. Interferences

#### E.1 Image periphery

**Figure 10:**
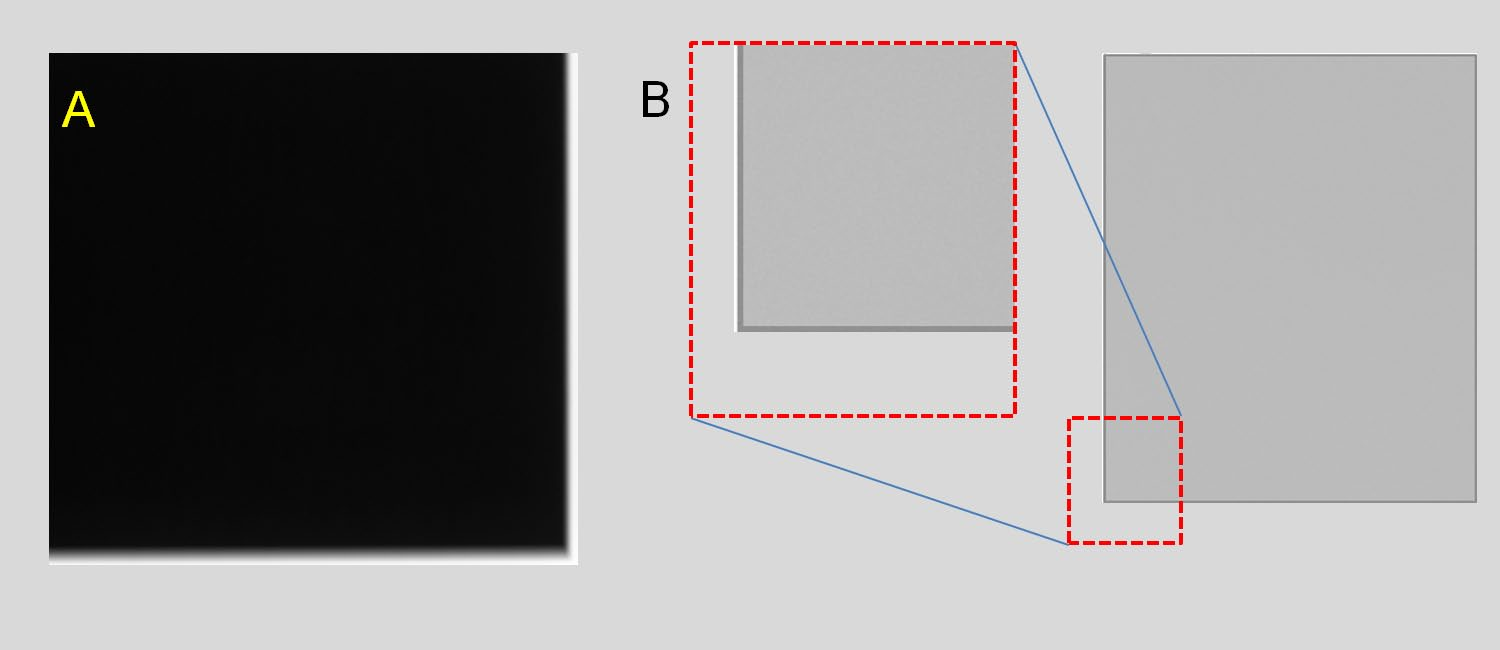
Artifacts associated with the periphery of images: (a) misalignment of collimator (GE XR656); (b) edges padded with maximum pixel value in the image harvested from Carestream Revolution mobile system.

The periphery of a flat‐field image can be affected by penumbra, scatter, collimator misalignment (Fig. 10(a)) and artificially filled‐in pixels (“padding,” Fig. 10(b)).^3^, ^8^ An example of the last type occurs in images harvested from a Carestream Revolution mobile system. The system crops the four edges during postacquisition processing to produce “for‐presentation” images but pads these edges with the maximum available value in harvested “for‐processing” images (IEC nomenclature – ORIGINAL DATA). These extreme pixel values on edges can greatly increase the values of nonuniformity and anomalous pixels metrics when they are included for the analysis, even if the central field of view is fairly uniform.

#### E.2 Structured noise

**Figure 11:**
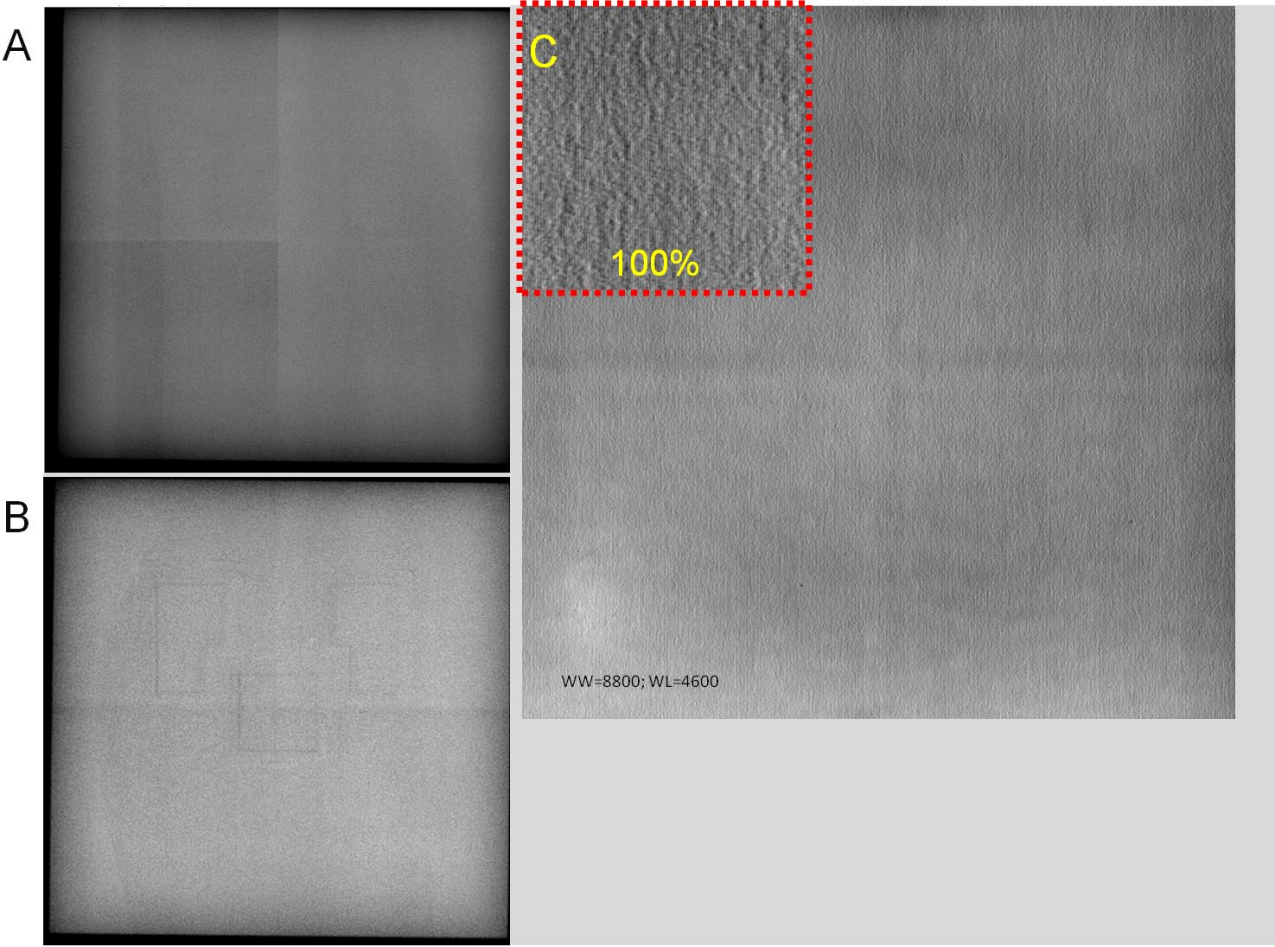
Examples of structured noise: (a) improperly calibrated detector (Siemens Aristos); (b) AEC cells visible in a flat‐field image (Siemens Aristos); (c) grid lines in a system with fixed grid (GE XQi).

Structured noise could adversely affect the detector nonuniformity metrics.^(3)^ An improperly calibrated image may show structured noise such as detector blocks and the outline of Automatic Exposure Control (AEC) chambers (Fig. 11(a) and (b)). These images should be excluded during the artifact evaluation and a service call should be placed for the detector. Some systems have fixed grids (e.g., GE Revolution XQ/i). The grid lines as well as the detector structure might appear in high exposure flat‐field images (Fig. 11(c)), although they were not as noticeable with low exposure. Performing nonuniformity analysis on the subtraction of two images at the calibration condition is recommended by TG‐150 for this scenario. However, images should be carefully evaluated for correlated noise since the subtraction will also eliminate them and result in a biased nonuniformity measurement.

#### E.3 Detector position relative to the A‐C axis at calibration

**Figure 12:**
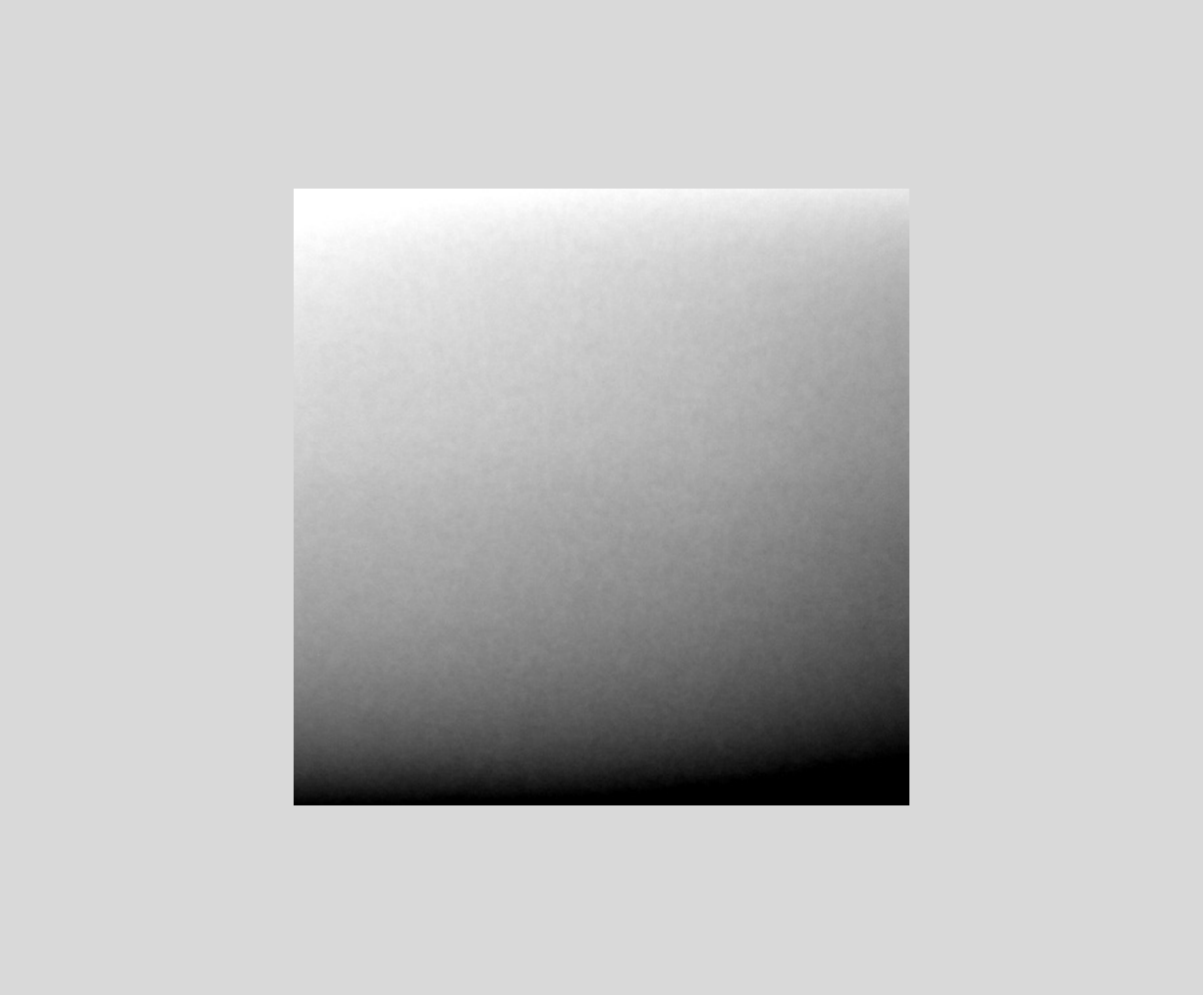
Flat‐field acquired in reversed calibration orientation (GE XR656).

Our results demonstrate that the relationship between the A‐C orientation of the detector during calibration and its orientation during testing can affect the nonuniformity metric. This is not only an issue for the Carestream DRX wireless detectors, but for any cassette‐based detectors that are not permanently integrated into the detector support assemblies including the GE Discovery XR656 wireless detectors. Because the detectors can be placed in the reversed calibration orientation (worst‐case scenario) the gradient from the heel effect can be doubled (Fig. 12). We found that the magnitude of the gradient was 33% from one edge of the detector to the other, which was large enough to elicit complaints from radiologists viewing clinical images. The nonuniformity values, especially global nonuniformities for noise and SNR, increased as much as 186% from this effect (see Det2 Table Bucky vs. cross‐table position, Table 6).

#### E.4 Image lag

**Figure 13:**
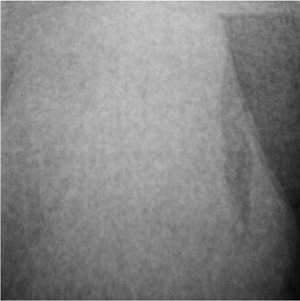
Lag image shown in a flat‐field image (GE XR656).

Another phenomenon affecting nonuniformity is image lag. If the flat‐field images are acquired immediately after acquisition of images with high contrast objects or background, such as lateral chest radiography with overexposed background (Fig. 13), the lag image will appear in the flat‐field images. Without appropriate caution, inverse lag images can be burned into the gain and offset correction maps when detector calibration is performed shortly after the acquisition of images with high signal levels. In this scenario, the values of nonuniformity and anomalous pixel metrics are artificially increased.

#### E.5 Saturation

**Figure 14:**
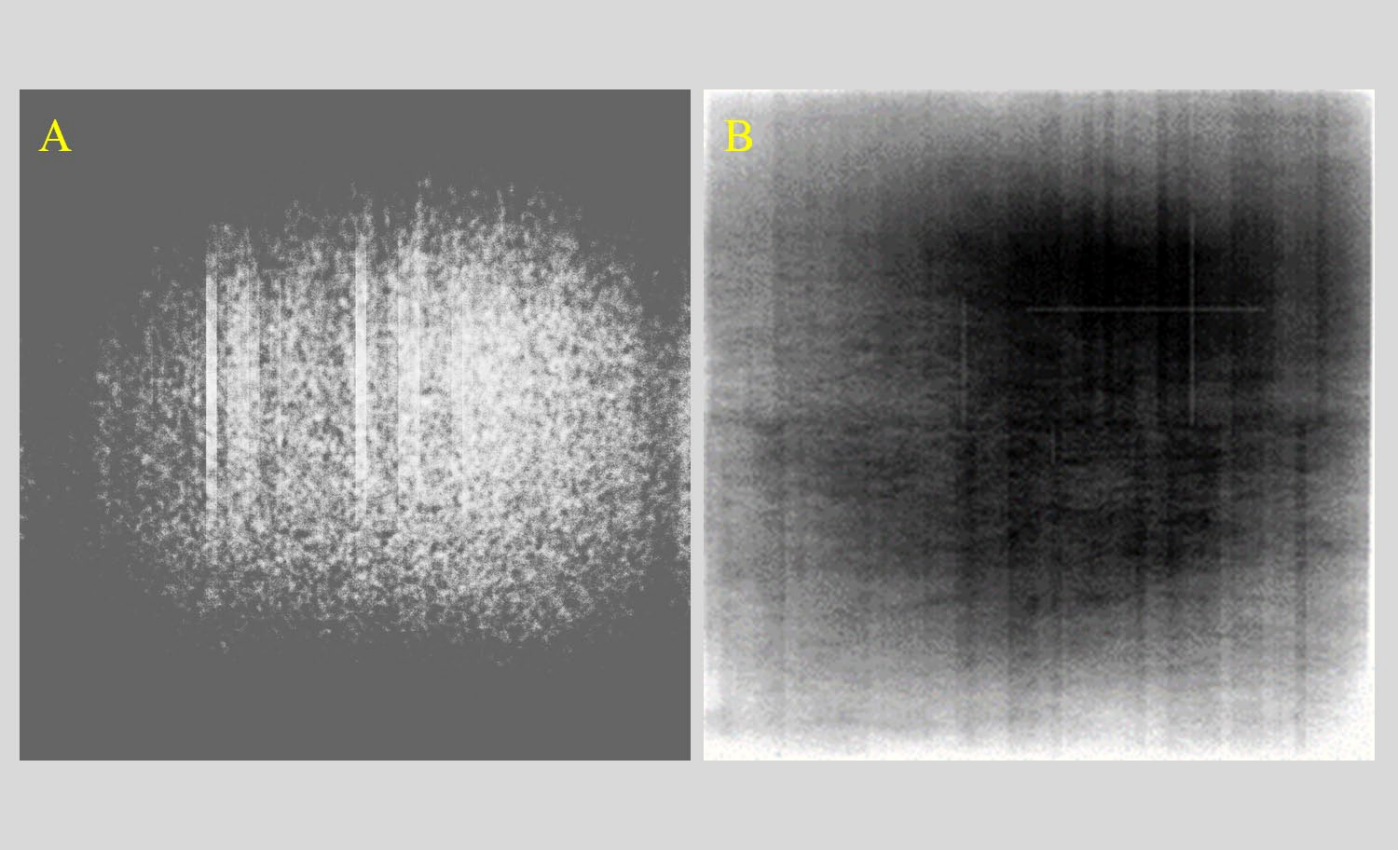
Partially saturated image (a) with structured noise (GE XR656). Flat‐field uncorrected (b) for gain, with offset on right for comparison (GE XQi).

TG‐150 suggested exposure levels for the additional flat‐field exposures relative to the calibration condition. These exposure levels worked well for all systems that we tested, except the GE XR656. The calibration condition for the XR656 was increased to 40 mAs for the wall stand position with SID=180 cm. When the mAs was doubled, some of the detectors were either partially or fully saturated (Fig. 14). In this scenario, the detector response will be skewed. Although the large signal capacity test recommended by TG‐150 was not performed in our limited implementation, which only evaluated nonuniformity metrics from images with high exposures, these metrics would also be adversely affected. Therefore, care should be exercised to ensure that the mAs selected for the high exposure flat‐field image does not saturate the detector.

## IV. DISCUSSION

Images should be carefully inspected before performing quantitative analyses since the presence of artifacts can render these analyses ineffective. Dave et al.^(3)^ reported that anomalous pixel detection would fail if an unusually large number of defects or structured noise was present, leading to a large standard deviation in an ROI such that the criteria of mean ±⋅σ for anomalous pixels is no longer sufficiently sensitive. Lag images, temporary signals from past exposures, are superimposed on subsequent acquisitions such that quantitative measurements do not reflect true detector performance. Similarly, if the test images do not contain the entire field of view because of errors in autopositioning, misaligned collimation, excessive edge padding, or penumbra, the metrics do not correctly represent detector performance, either. Instead, they suggest system malfunctions. Consequently, their presence affects the quality of diagnostic images and, therefore, eventually may affect diagnosis. Thus, inspecting images carefully should be the first step of assessing the test images and, if unusual artifacts are identified, testing should be repeated only after the system is corrected.

Nitrosi et al.^(11)^ have published image quality metrics for detectors from four different manufacturers including older DR systems from Carestream and GE. Their work was based on a quality control protocol for direct DR systems advanced by the Italian Association of Physicists in Medicine or AIFM.^(16)^ Their metrics included local and global nonuniformity of signal and SNR with definitions similar to TG‐150. The numerators of their metrics are identical to TG‐150 metrics in (2), (4), (5), (7), however the denominators are different. The local nonuniformities are normalized to the local ROI mean rather than the global ROI mean. The global nonuniformities are normalized to the range of ROI means instead of the global ROI mean. Both differences tend to make the numerical values of the metrics smaller. Compared with our TG‐150 measurements, their values are in the same order of magnitude as ours, despite differences in hardware and beam quality when the detectors are properly calibrated. However, when the detectors were miscalibrated, TG‐150 reported local SNR nonuniformity of 30%∼ 50% and global SNR nonuniformity of 40%∼ 80%, which were much higher than the 4.6%±1% and 15.5%±1% reported for these metrics by Nitrosi et al. Hence, it seems that the detector miscalibration affects TG‐150 metrics of SNR nonuniformity more than AIFM's.

MTF values determined by TG‐150 testing were consistent with studies from other groups. One group recently reported MTF about 20% at spatial frequency of 2.5 lp/mm with GE FlashPad and Carestream DRX‐1C,^(17)^ compared to approximately 22% in our results. The same group also reported 1.25 lp/mm and 2.55 lp/mm for 50% and 20% MTF with Carestream DRX‐1C detectors using a RQA5 beam with a 0.5 mm Cu +1.0 mm Al filter,^(16)^ which were similar to 1.1 lp/mm and 2.6 lp/mm at the same MTFs measured in our study, despite a difference in kVp (70 vs. 80 kVp).

The image quality metrics varied with the ROI sizes, as indicated by 3, 4. The same metric varied up to threefold across the four sets of ROIs. For example, the median of global SNR nonuniformity for the 64×64 ROIs was 14.2% while the median for the 256×256 ROIs was 4.2%. The metrics for nonuniformity were usually higher for a smaller set of ROIs because extreme pixel values had a greater influence on the mean and variance when the ROIs were smaller. For the same reason, the minimum SNR was lower for a smaller set of ROIs. In the optimum case, the ROIs would be sized and aligned to coincide with the electronic components reading out the detector elements so that the metrics would be representative of specific portions of the detector subassembly.

The positioning tests showed that detector orientation affects all of the image quality metrics except for the MTF. After the uncertainty of orientation was ruled out, the repeated measurements were consistent with the ones at the exact calibration location; however, the variation in repeated measurements for the same detector from repositioning (Fig. 5) was greater than or equal to the variation among seven different detectors tested at the exact calibration location (Table 4). Repositioning not only introduced additional uncertainty, the repeated measurements were always worse than those measured at the exact calibration location.

Performing the tests immediately after calibration in the exact same location yields the optimum results and should ideally constitute the baseline values for testing. However, since the exact exposure location is unlikely to be reproduced unless the wireless detector is held in an exposure station and the X‐ray tube is automatically repositioned to the proper location, subsequent tests are always going to be disadvantaged. These values may be misinterpreted as indicative of degraded performance. For the purpose of establishing a baseline, it may be wiser to use the mean of several repeated measurements after repositioning. Subsequent measurements to assess constancy of performance are more likely to reflect the mean than the optimum results at the exact calibration position. This problem is particular to the cassette‐based DR detectors that do not have exposure position controls, such as bedside or tabletop applications, rather than the integrated DR detectors.

Conversely, the problem for integrated DR detectors and cassette‐based detectors with position controls is that autopositioning must be accurate and reproducible. Otherwise, test metrics are neither accurate nor precise.

The metrics reported by both TQT and TG‐150, in general, agree with each other. However, because the size of the ROIs and positioning affect the measurement, caution should be taken when comparing their numerical values.

The metrics reported by both QAP and TG‐150 agreed with each other for local and global signal nonuniformity measurements, but not for SNR nonuniformity and MTF. Two possible causes exist for lower global SNR nonuniformity values reported by QAP. First, QAP measured noise by the subtraction of two flat‐field images at the calibration condition, while, TG‐150 measured noise using only one image at the calibration condition. The subtraction eliminates the contribution of structured noise and gradients, and would result in lower SNR nonuniformity, as we observed. Second, the ROI size, as illustrated with the DRX‐1C detectors, might have affected the results. The ROIs used by QAP for the three nonuniformity metrics were larger (Table 2, 150×150) than those used by our implementation of TG‐150 (128×128). Our observation of the QAP measurements agreed with our findings with the DRX‐1C (3, 4) — larger ROIs yield lower values for nonuniformity. Further investigation is needed to determine the relative magnitude of these effects.

Although MTF measured by TG‐150 tests and QAP were highly linearly correlated ($R_{2}$ 0.95), their absolute differences were as much as ∼ 24%. The difference in the methods of measuring MTF (TG‐150's bar pattern method versus QAP's edge method) was unlikely to be the cause because TQT also used the edge method and its MTF values agreed with those measured by the TG‐150 bar pattern method. In the case of the QAP, the lower MTF reported by the TG‐150 method might be explained by the difference in the beam quality between the two methods. The TG‐150 test used a 20‐mm aluminum filter to harden the beam to the calibration condition, which decreased the contrast compared to the unfiltered beam used in the QAP exposures of the IQST (aka, MTF phantom) for MTF measurements.

## V. CONCLUSIONS

This study shows that the values of common image metrics employed by TG‐150 and TQT, such as nonuniformity, minimum SNR, and MTF, are consistent. Comparisons between TG‐150 and QAP indicate that their local and global signal nonuniformity values are similar and correlated, but not their SNR nonuniformity and MTF values. The inconsistency might be explained by the differences in methods, beam quality, and ROI used by the QAP and TG‐150 tests. However, this study suggests that TG‐150's SNR nonuniformity metrics are more sensitive to detector nonuniformity, compared to the QAP. The study also suggests that same ROI size should be used for consistent results since nonuniformity metrics and minimum SNR are dependent on ROI sizes. Detector testing at the exact calibration location after calibration would reduce uncertainty, but if this is not feasible, repeated measurements should be performed to establish a baseline. All images should be carefully reviewed before analyses to avoid any nonuniformity contributed by artifacts. Overall, TG‐150 provides a good guide for digital detector testing, but the testing and analysis should be performed in a consistent manner.

## ACKNOWLEDGMENTS

The authors would like to express their gratitude to Dr. Eric Gingold and the members of the Image Receptor Subgroup of TG‐150 for their development of the proposed tests, Dr. John Yorkston for helpful discussions on TQT test results, Dr. John Sabol for helpful information and discussions on the GE QAP test, and Dr. R. Benton Pahlka and Steve Bache for their help with data collection.

## COPYRIGHT

This work is licensed under a Creative Commons Attribution 3.0 Unported License.

## References

[1] Schreiner‐Karoussou A . Review of image quality standards to control digital x‐ray systems. Radiat Prot dosimetry. 2005;117(1‐3):23–25. 16464831 10.1093/rpd/nci722

[2] Greene T , Nishino T , Willis C . Independent implementation of AAPM TG‐150 draft image receptor test recommendations [abstract]. Med Phys. 2012;39(6):3648. 10.1118/1.4734819 28517639

[3] Dave J , Gingold E , Yorkston J , et al. Detecting anomalous pixels and correlated artifacts in digital detectors from flat‐field images [abstract]. Med Phys. 2012;39(6):3648. 10.1118/1.4734820 28517660

[4] Dave J , Gingold E , Yorkston J , et al. Initial implementation and evaluation of AAPM TG‐150 draft image receptor non‐uniformity testing recommendations. Med Phys. 2012;39(6):3648. 10.1118/1.4734818 28517653

[5] Jabri KN , Uppaluri R , Xue P . Management of pediatric radiation dose using GE's Revolution digital radiography systems. Pediatr Radiol. 2004;34(Suppl 3):S215–S220. 15558264 10.1007/s00247-004-1272-y

[6] Belanger B , Boudry JM , Katakam A , Palkus VS . Advances in and specifications for fluoroscopic imaging systems. In: Goldman LW, Yester MV, editors. Specifications, performance evaluation, and quality assurance of radiographic and fluoroscopic systems in the digital era. Madison, WI: Medical Physics Publishing; 2004 p. 69–102.

[7] X‐ray detectors [product information]. Waltham, MA: Perkin Elmer; 2015; Available from: http://www.perkinelmer.com/

[8] Droege RT . A practical method to routinely monitor resolution in digital images. Med Phys. 1983;10(3):337–43. 6877181 10.1118/1.595263

[9] Bencomo J , Le Duc T , McCraw F , Fallone B . Logit analysis of screen/film modulation transfert function data. J Imaging Sci. 1986;30(6):270–73.

[10] Christodoulou EG , Goodsitt MM , Chan H‐P , Hepburn TW . Phototimer setup for CR imaging. Med Phys. 2000;27(12):2652–58. 11190947 10.1118/1.1319522

[11] Nitrosi A , Bertolini M , Borasi G , et al. Application of QC_DR software for acceptance testing and routine quality control of direct digital radiography systems: Initial experiences using the Italian Association of Physicist in Medicine Quality Control Protocol. J Digit Imaging. 2009;22(6):656–66. 18769968 10.1007/s10278-008-9150-z PMC3043728

[12] Samei E . Performance of digital radiographic detectors: quantification and assessment methods. Advances in digital radiography. RSNA Categorical Course in Diagnostic Radiology Physics 2003; pp 37–47. Oakbrook, IL: RSNA; 2003.

[13] Bonferroni CE . Teoria statistica delle classi e calcolo delle probabilita. Libreria internazionale Seeber; 1936.

[14] Dunn OJ . Multiple comparisons among means. J Am Stat Assoc. 1961;56(293):52–64.

[15] Holman JP W. J. Gajda J. Experimental Methods for Engineers, 5th edition Columbus, OH: McGraw‐Hill; 1989.

[16] Samei E , Murphy S , Christianson O . DQE of wireless digital detectors: Comparative performance with differing filtration schemes. Med Phys. 2013;40(8):081910. 23927324 10.1118/1.4813298

[17] Wells J , Christensen J , Samei E . A consumer report for mobile digital radiography: a holistic comparative evaluation across four systems [abstract]. Med Phys. 2015;42(6):3720.

